# Malignant Mesothelioma Mimicking Invasive Mammary Carcinoma in a Male Breast

**DOI:** 10.1155/2015/298523

**Published:** 2015-09-10

**Authors:** Mohamed Mokhtar Desouki, Daniel Jerad Long

**Affiliations:** Department of Pathology Microbiology and Immunology, Vanderbilt University School of Medicine, Nashville, TN 37232, USA

## Abstract

Malignant mesothelioma is an uncommon tumor with strong association with asbestos exposure. Few cases of malignant pleural mesothelioma metastatic to the female breast have been reported. Herein, we presented, for the first time, a case of locally infiltrating malignant pleural mesothelioma forming a mass in the breast of a male as the first pathologically confirmed manifestation of the disease. Breast ultrasound revealed an irregular mass in the right breast which involves the pectoralis muscle. Breast core biopsy revealed a proliferation of neoplastic epithelioid cells mimicking an infiltrating pleomorphic lobular carcinoma. IHC studies showed the cells to be positive for calretinin, CK5/6, WT1, and CK7. The cells were negative for MOC-31, BerEp4, ER, and PR. A final diagnosis of malignant mesothelioma, epithelioid type, was rendered. This case demonstrates the importance of considering a broad differential diagnosis in the setting of atypical presentation with application of a panel of IHC markers.

## 1. Introduction

Malignant mesothelioma is an uncommon tumor with a high mortality rate. The reported incidence of mesothelioma in the United States is approximately 3,300 cases per year [1]. Asbestos exposure, mostly occupational, is a well-known risk factor associated with pleural mesothelioma [2]. Other contributing factors which may be associated with increased incidence of mesothelioma include external radiation at nuclear facilities and exposure to Thorotrast which is a radioactive contrast agent used in diagnostic radiologic procedures among other factors [3, 4]. The incidence of mesothelioma is now declining, which is likely due to significant measures taken to limit asbestos exposure [5].

Metastasis of malignant mesothelioma to distant organs has been reported to the central nervous system, the chest, abdominal and pelvic walls, oral cavity and tongue [6–8]. A case of metastatic malignant pleural mesothelioma to a 51-year-old female breast has been also reported [9]. To the best of our knowledge, there is no reported metastatic or locally extended malignant mesothelioma to a male breast in the English literature. Herein, we presented, for the first time, a case of locally infiltrating malignant pleural mesothelioma forming a 5 cm mass in the right breast of a 77-year-old man as the first pathologically confirmed manifestation of the disease.

## 2. Case Report

### 2.1. Clinical Presentation

A 77-year-old male presented with palpable abnormality on the right breast. The past medical history was significant for a recent complicated parapneumonic effusion requiring right thoracotomy and pleural decortication with benign pathologic findings eleven months prior to presenting with the breast mass. The patient also had a history of squamous cell carcinoma and melanoma in situ of the skin. The patient reported an occupational history of asbestos exposure. Family history was significant for breast cancer and BRCA1 mutation positivity in two siblings; the patient's own BRCA status was unknown. On physical examination, there was a fixed, firm mass in the right breast measuring approximately 5 cm in greatest dimension.

### 2.2. Imaging Studies

**Figure 1:**
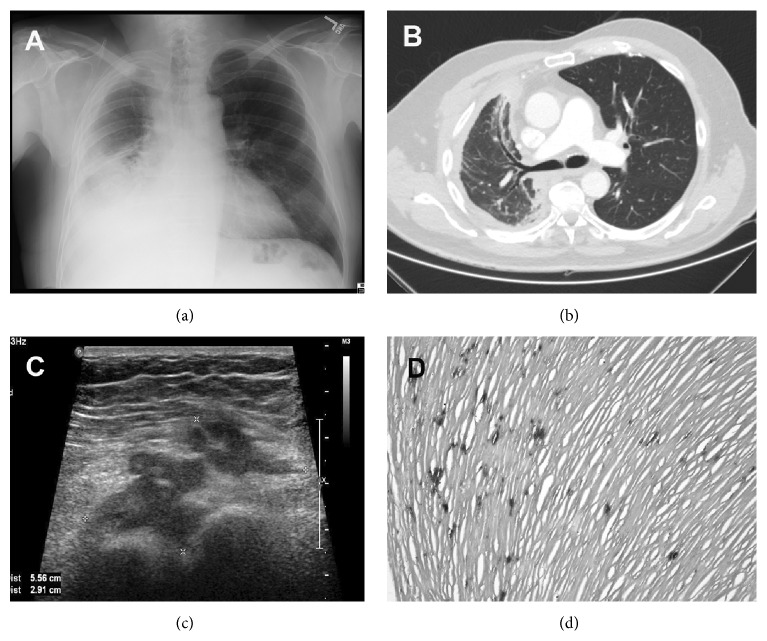
(a) Anteroposterior chest X-ray shows pleural thickening along the right lateral chest wall and blunting of the right costophrenic angle. (b) A chest CT scan shows extensive pleural thickening on the right side and calcified pleural plaque on the left side. (c) A breast ultrasound shows an irregular, hypoechoic mass measuring 5.6 × 2.9 × 3.6 cm. A portion of the mass involves pectoralis muscle and extends into the intercostal muscles. (d) Pleural biopsy shows plaque formation with dense fibrosis, minimal inflammation, and dystrophic calcification with no evidence of malignancy.

Anteroposterior chest X-ray showed pleural thickening along the right lateral chest wall and blunting of the right costophrenic angle (Figure 1(a)). A chest CT scan with contrast demonstrated a contracted right hemithorax with an irregular pleural-based process that extends through the intercostal muscle and into the subcutaneous adipose tissue indicating direct spread rather than a metastasis in breast tissue. Bronchiectasis of right middle and lower lobes, right middle lobe atelectasis, and prior granulomatous disease have been also reported (Figure 1(b)). A diagnostic breast mammogram revealed predominantly fatty breast parenchyma and no morphologically abnormal lymph nodes in the axilla. A diagnostic breast ultrasound revealed an irregular, hypoechoic mass in the right breast with angular margins measuring 5.6 × 2.9 × 3.6 cm. A portion of the mass appeared to involve the pectoralis muscle and possibly extended into the intercostal muscles (Figure 1(c)). Fine needle biopsy was recommended.

### 2.3. Histopathology

**Figure 2:**
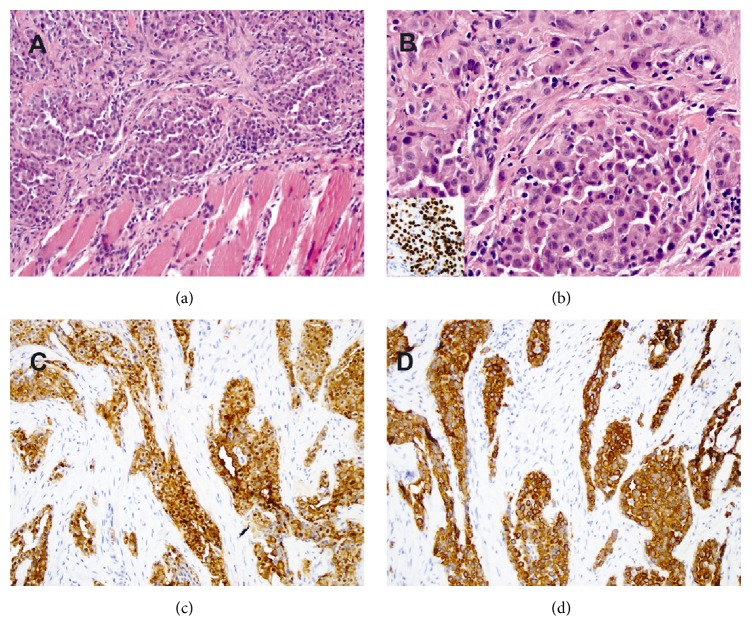
Histopathology and immunoprofile of the metastatic malignant mesothelioma to the breast. ((a) and (b)) Representative H&E captions from the metastatic malignant mesothelioma in the breast biopsy which show proliferation of neoplastic epithelioid cells forming cords and nests which infiltrate the breast parenchyma and skeletal muscles. The neoplastic cells are round to polygonal with moderate cytoplasm, moderate cytologic pleomorphism, and occasional nucleoli. The neoplastic cells are positive for WT1 (inset in (b)), calretinin (c), and CK5/6 (d).

Biopsy from the right pleural decortication described grossly as a fragment of red-tan tissue measuring 0.9 × 0.7 × 0.2 cm was entirely submitted in one cassette. Microscopic examination was performed at an outside facility and reviewed by expert lung pathologists in consensus at our institution subsequent to the diagnosis of the breast lesion and reported as pleural plaque with dense fibrosis, minimal inflammation, and dystrophic calcification with no evidence of malignancy (Figure 1(d)). Breast needle core biopsy revealed a proliferation of neoplastic epithelioid cells in cords and nests infiltrating breast parenchyma and skeletal muscles. The neoplastic cells were round to polygonal in shape with moderate cytoplasm, moderate cytologic pleomorphism, and occasional nucleoli mimicking an infiltrating pleomorphic lobular carcinoma (Figures 2(a) and 2(b)). There were focal gland-like and micropapillary structures. Rare mitotic activity was present.

### 2.4. Immunohistochemistry (IHC)

IHC studies showed the tumor cells to be strong and diffusely positive for WT1 (inset in Figure 2(b)), calretinin (Figure 2(c)), CK5/6 (Figure 2(d)), and CK7. The cells were negative for MOC-31, BerEp4, ER, PR, S100 protein, and HMB-45. Based on the morphologic and IHC findings, a final diagnosis of malignant mesothelioma, epithelioid type, was rendered.

## 3. Discussion

Malignant neoplasms of the male breast, whether primary tumors or metastases from distant sites, are rare. In the United States, the incidence of male breast carcinoma is approximately 1.3 per 100,000 [10], and metastases account for approximately 1.3–2.7% of all malignant breast tumors [11]. In this reported case, there were clinical findings concerning both primary mammary carcinoma (clinically palpable breast mass, family history of breast cancer and BRCA1 mutation, and recent benign pleural biopsy) and malignant mesothelioma (history of asbestos exposure and pleural thickening). Given the previous thoracotomy and the predilection of mesothelioma to spread through surgical and drainage sites [12], there is a possibility that this might be causing the chest wall and breast involvement. The morphologic overlap between epithelioid variants of malignant mesothelioma and adenocarcinoma is well described [13]. This overlap is exemplified in this case, with the malignant mesothelioma forming infiltrating nests, cords, and occasional gland-like structures, which impart an overall histologic akin to intermediate grade invasive pleomorphic lobular carcinoma.

Clinical history and morphologic features of a tumor are critically important in the determination of primary versus secondary origin of a breast tumor. However, in cases where the clinical and morphologic data is inconclusive, similar to this reported case, an IHC evaluation may be useful. Approximately 20–25% of mammary cancers are negative for ER, and tumors with a high nuclear grade have the highest proportion of ER-negativity [14]. In the reported case, the morphology and the negativity for ER and PR in the tumor cells raised the suspicion of an extramammary malignancy, given the tumor's intermediate grade appearance. Although CK7 is positive in greater than 90% of mammary carcinomas [15], many other tumors express CK7, including malignant mesothelioma, as in this case [13]. Another word of caution is the utilization of calretinin which is a known marker for mesothelioma. Approximately 15% of breast carcinomas stain positive for calretinin. These tumors are more likely to be ER- and high-grade tumors of the basal-like phenotype [16]. Therefore, we recommend utilizing a full panel of IHC markers when evaluating the possibility of an extramammary metastasis.

In the reported case, the correct diagnosis of malignant mesothelioma was important, given the significant difference in management between patients with mammary carcinoma and those with malignant mesothelioma. For example, primary surgery is typically considered in patients with nonmetastatic mammary carcinoma, whereas surgical resection is only performed in a subset of patients with malignant pleural mesothelioma. Additionally, there are significant differences between the chemotherapy regimens and radiation therapy protocols utilized for patients with these malignancies [17].

In conclusion, this case of malignant mesothelioma forming a mass lesion, as the first pathologically proven manifestation in a male breast, demonstrates the importance of considering a broad differential diagnosis in the setting of a rare tumor or atypical presentation. When there is concern for an extramammary metastasis/local spread, a panel of IHC markers may be helpful, and malignant mesothelioma should be considered among the potential neoplasms.
